# Impact of Pain and Palliative Care Services on Patients

**DOI:** 10.4103/0973-1075.78446

**Published:** 2011

**Authors:** S Santha

**Affiliations:** Post Graduate Department of Commerce and Research Centre, St. Peter’s College, Kolenchery, Ernakulam Dist., Kerala, India

**Keywords:** Friedman repeated measures analysis of variance on ranks, Hospice, Neighborhood network in palliative care, Palliative care, PPC units

## Abstract

**Background:**

Palliative care has become an emerging need of the day as the existing health-care facilities play only a limited role in the care of the chronically ill in the society. Patients with terminal illness in most cases spend their lives in the community among their family and neighbors, so there is the need for a multi disciplinary team for their constant care. Volunteers are primary care givers who originate normally from the same locality with local knowledge and good public contact through which they can make significant contributions in a team work by bridging the gap between the patient community and outside world.

**Aim:**

The present study has been undertaken to analyze the impact of palliative care services on patients by considering 51 variables.

**Materials and Methods:**

The respondents of the study include 50 pain and palliative care patients selected at random from 15 palliative care units functioning in Ernakulam district. The analysis was made by using statistical techniques viz. weighted average method, Chi-square test, Friedman repeated measures analysis of variance on ranks and percentages.

**Results:**

The study revealed that the major benefit of palliative care to the patients is the reduction of pain to a considerable extent, which was unbearable for them earlier. Second, the hope of patients could be maintained or strengthened through palliative care treatment.

**Conclusion:**

It is understood that the services of the doctors and nurses are to be improved further by making available their services to all the palliative care patients in a uniform manner.

## INTRODUCTION

Palliative care is a holistic care which fulfills the requirements of chronically ill patients. Those who need continued supportive care spend their lives not in the hospital, but in the community among their family and neighbors. Hence, the community has a major role in the care of these individuals. Yuen *et al*.,[1] in their study “Palliative care at home: general practitioners working with palliative care teams” stated that home care was the preferred option for most people with a terminal illness, and providing home care relies on good community-based services, a general practice workforce competent in palliative care practice, and willing to accommodate patients’ need. Devi, *et al*.,[2] in a study, “Setting up home-based palliative care in countries with limited resources: a model from Sarawak, Malaysia,”, described the set up of a home-care program in Sarawak (the Malaysian part of the Borneo Island), where half the population lives in villages that are difficult to access. The program had been sustainable and cost efficient, serving 936 patients in 2006. The results showed that pain medication could be provided even in remote areas with effective organization and empowerment of nurses, who were the most important determinants for the set up of this program. Zerzan *et al*.,[3] in their study, “ Access to Palliative Care and Hospice in Nursing Homes”, stated that hospice improves end-of-life care for dying nursing home residents by improving pain control, reducing hospitalization, and reducing use of tube feeding, but it is rarely used. Hospice use varies by region, and rates of use are associated with nursing home administrators’ attitudes toward hospice and contractual obligations.

Data show that 80% of all palliative care services in the country are delivered in Kerala, reaching 30% of the needy patients, whereas these services reach only to 2% in India. Kerala’s attempts at caring for terminally ill patients have been regarded as a model for the rest of the world. Kerala Government is the only State Government in Asia which has introduced a palliative care policy in the State for the first time. The Neighborhood Network in Palliative Care (NNPC) is a volunteer-driven movement that has gained momentum in Kerala, especially in Malabar Region, where the volunteers are the arms of the community, supporting the patient in collaboration with governmental and nongovernmental agencies in Kerala.

### Significance of the study

Palliative care is a prerequisite for a complete medical care. It provides the best care to the patients and their families. In India, the present medical and hospice systems do not have the capacity to guarantee quality of life for the majority of people with life-limiting illnesses or for their care givers and survivors, which focuses upon the identification and control of observable and predictable physical symptoms. The existing healthcare facilities are more attuned to caring for acute health problems and they play only a limited role in the care of the chronically ill in the society. Patients with terminal illness need a multidisciplinary team and constant care. This has lead to a mounting need for palliative care.

Pain and palliative care units (hereafter referred as PPC Units) are committed to being responsible centers of the communities where they operate by making a visible impact on billions of lives across the world with their renowned products. They also touch the lives of those who are in need of care and attention. In Kerala, the tremendous developments made in palliative care in the State have made the end-of-life phase of the terminally ill more bearable. Palliative care remains the only and indeed the most appropriate form of treatment for the patients presenting at incurable stages. There is a need to advocate adequate policy development and effective program implementation in the area of palliative care. Moreover, the review of earlier literature revealed that most of the studies in palliative care have been conducted in the field of medical science. No study has so far been conducted for analyzing the impact of palliative care services on patients. In this context, the present topic entitled “Impact of Pain and Palliative Care Services on Patients” assumes greater importance.

### Scope of the study

The present study has been undertaken to analyze the impact of palliative care services on the patients availing such services. The assessment has been made by considering the perception of patients in Kerala. However, the focus of the study is the palliative care patients of Ernakulam District, Kerala.

### Objective of the study

The main objective of the study is to know the impact of palliative care services on the patients availing such services in Kerala.

### Hypothesis of the study

H_O__1_There is no difference in the level of satisfaction among the PPC patients with regard to services of medical professionals in Kerala.

### Selection of sample

The PPC patients have been selected from the data base maintained by the PPC units of the Ernakulam district, Kerala State. There are in all 22 PPC units functioning in Ernakulam district as on July 31, 2009. Only 15 units in Ernakulam are offering home care services. Convenience sampling method was adopted for selection of sample. A sample of 50 patients (of 2000 patients) was selected from these 15 units for the purpose of study.

### Collection of data

The data required for the study were collected from both primary and secondary sources. The primary data were collected from the respondents based on structured questionnaire. The secondary data were collected from reports, books, and journals published by the consortium of PPC Units in Ernakulam District, Institute of Palliative Medicine, and from various web sites.

### Tools of analysis

For the purpose of analysis, statistical tools like averages, percentages, rank test, and Friedman repeated measures analysis of variance on ranks were used. To study the level of satisfaction in the palliative care services among patients in Kerala, the relevant questions were asked in five point scale with the following options: highly satisfied, satisfied, not satisfied, dissatisfied, and no opinion. These questions were scored in the order of magnitude from 5 to 1. Overall score of each respondent was found out, which form the basis for comparison. The Friedman repeated measures analysis of variance on ranks (nonparametric test) was used to compare the effects of a series of different experimental treatments on a single group. Each subject’s responses were ranked from smallest to largest without regard to other subjects, and then the rank sums for the treatments were compared.

### Period of the study

The study covers a period of six months, that is, from July 2009 to January 2010.

### Impact of palliative care service – Analysis

For analyzing the impact of PPC services on patients, 51 variables have been considered. The study revealed that the majority of the patients (52%) under palliative care treatment were men in the age group of above 60 years. It is understood that in Ernakulam District, palliative care is mainly provided to cancer patients because 50% of the beneficiaries of palliative care services are cancer patients. Table 1 reveals that the major physical problem of the patients is pain and the problem of incontinence is ranked as second. The Friedman Chi-square test result [Table 2] revealed that there is significant difference in the type of physical problems faced by the patients of different age groups (Chi-square = 345.495 with 22 d.f.at 1% level). Most of the patients are not able to stay in their job and their children could not continue their schooling because of their illnesses which they ranked as the major social problem, and they also have fear on account of illness [Table 3]. Huge medical expenditure is the major financial problem faced by the patients, followed by intractable debt which is ranked as second [Table 4]. The major medical care provided by the doctors is prescribing medicines. They also help the patients to reduce their sufferings through touch and closeness [Table 5]. Most of the patients, irrespective of their age, are either highly satisfied or satisfied with the services of the doctors [Table 6]. Chi-square test result [Table 6] revealed that there is no significant relationship between the age of the patients and their level of satisfaction in the services of the doctors (Chi-square = 1.531 with 1d.f. at 5% level). The major service provided by the nurses is attending to the bed sore of the patients. They also give medicines to the patients as per the directions of the doctors, which is ranked as second by the patients [Table 7]. It is revealed that all the patients in the age group of 20 to 40 years are highly satisfied and patients in the age group of above 40 years are either highly satisfied or satisfied with the services of nurses [Table 8]. There is no significant relationship between the age of the patients and their level of satisfaction in the services of the nurses (Chi-square = 0.045 with 1 d.f. at 5% level) [Table 8]. The Chi-square test results given in Table 6 and Table 8 revealed that there is no significant difference in the level of satisfaction among the patients with regard to the services of doctors and nurses. Therefore, the null hypothesis (H_O__1_) stating that there is no difference in the level of satisfaction among the PPC patients with regard to services of medical professionals in Kerala stands accepted.

**Table 1:** Type of physical problems faced by the patients

Physical problem	Mean	Rank
Pain	21.868	1
Breathlessness	20.538	4
Fatigue	20.550	3
Drowsiness	19.587	6
Insomnia	19.333	8
Dehydration	18.200	17
Constipation	19.133	11
Anorexia	19.830	12
Nausea	17.000	20
Physical losses	18.333	16
Edema	18.857	13
Incontinence	20.929	2
Loss of function	18.000	18
Vomiting	19.833	5
Bed sores	18.833	14
Loss of mobility/dependency	19.250	9
Fumigating wounds	17.500	19
Disfigurement	19.167	10
Difficult to swallow	19.444	7
Itching	18.500	15

Source: Primary data

**Table 2:** Type of physical problems of patients and age of the patients (Friedman repeated measures analysis of variance on ranks)

Category	20–30		30–40		40–50		50–60		Above 60 years	
	Mean	Rank	Mean	Rank	Mean	Rank	Mean	Rank	Mean	Rank
Pain	21.0000	2	22.0000	1	21.600	2	21.846	2	22.000	1
Breathlessness			21.0000	2	19.0000	7	21.0000	4	20.778	3
Fatigue			20.0000	3	20.250	6	20.714	5	20.625	4
Drowsiness			19.0000	4	19.0000	7	19.0000	8	20.500	5
Insomnia					17.500	8	19.0000	8	19.727	9
Dehydration			21.0000	2	21.0000	4	21.333	3		
Constipation					21.0000	4	19.143	7	18.857	12
Anorexia					20.500	5	18.333	9	19.000	11
Nausea							16.250	14	17.000	14
Physical losses					16.500	10	19.000	8	19.500	10
Edema			18.500	5	0.0000		17.667	12	21.000	2
Incontinence			22.0000	1	21.250	3	22.000	1	20.286	6
Loss of function	20.000	3	16.000	6						
Vomiting	22.000	1			22.000	1	18.000	10	19.000	11
Bed sores	18.0000	5					21.000	4	17.667	13
Loss of mobility/dependency	19.000	4	18.500	5	21.000	4	17.750	11	20.000	7
Fumigating wounds			20.000	3			16.500	13	17.667	13
Disfigurement			21.000	2	21.000	4	20.000	6	17.667	13
Difficult to swallow					17.000	9			19.750	8
Itching					16.500	10			20.000	7

Source: Primary data; χ^2^= 345.495 with 22 degrees of freedom. Significant at 1% level

**Table 3:** Type of social problems faced by the patients

Type of social problem	Mean	Rank
Not able to stay in my job/go to school	15.350	1
Social isolation	14.524	3
Not able to fulfill my prior role in the family/society	14.375	4
Sadness	13.350	10
Not able to be active in the society/community	14.318	5
Depression	14.111	7
Not able to keep up friendships	12.000	12
Anger	12.500	11
Anticipatory bereavement	10.167	14
Lack of social safety	14.167	6
Fear	15.125	2
No relatives available for help	13.923	8
Neglect	12.500	11
Change in faith/beliefs	13.714	9
Loss of social roles	11.600	13

Source: Primary data

**Table 4:** Type of financial problems faced by the patients

Type of financial problems	Mean	Rank
Poverty due to absence of income earning member	5.231	3
Huge medical expenditure	5.732	1
Children dropped out of school	4.000	5
Intractable debt	5.313	2
Family member gave up work due to illness	4.333	4

Source: Primary data

**Table 5:** Type of physical care provided by the doctors

Type of physical care	Mean	Rank
Medicines	4.946	1
Exercises and aids	3.625	4
Touch and closeness	4.000	2
Discussion between me and family members	3.692	3

Source: Primary data

**Table 6:** Age of the respondents and their level of satisfaction in the present services of the doctors

Age	Highly satisfied	Satisfied	No opinion	Total
20–30		1	-	1
30–40	1	1	1	3
40–50	2	5		7
50–60	6	5	2	13
Above 60 years	10	13	3	26
Total	19	25	6	49

Source: Primary data; χ^2^ = 1.531 with 1 degree of freedom. Not significant at 5% level

**Table 7:** Type of physical care provided by the nurses

Type of physical care	Mean	Rank
Bathing	7.0000	3
Attending to the bed sore	8.0000	1
Changing clothes	5.0000	7
Giving medicines	7.7050	2
Dressing the wounds	6.3333	5
Changing the “condom catheter”	6.8700	4
Training the family members in simple nursing tasks	5.8333	6

Source: Primary data

**Table 8:** Age of the respondents and their level of satisfaction in the present services of the nurses

Age	Highly satisfied	Satisfied	No opinion	Total
20-30	1	-	-	1
30-40	3	-	-	3
40-50	2	4	1	7
50-60	9	4		13
Above 60 years	17	8	1	26
Total	32	16	2	50

Source: Primary data; χ^2^ = 0.045 with 1 degree of freedom; Not significant at 5% level

“Attending to the bed sore” is the major physical care provided by the volunteers to the patients, which is ranked as first by the patients. Second, volunteers also help the patients to change clothes and give them medicines as prescribed by the doctor [Table 9]. The major psychological care provided by volunteers is chatting with the patients. Volunteers also listen to the sorrows of the patients, which is ranked as second by the patients [Table 10]. The major financial care provided by the volunteers is supply of medicines to the patients at free of cost. Second, they supply rice and provisions to the patient’s family [Table 11]. The major spiritual care provided by the volunteers to the patients is psychological boost [Table 12].

**Table 9:** Type of physical care provided by the volunteers

Type of physical care provided by the volunteers	Mean	Rank
Bathing	7.000	4
Attending to the bed sore	7.800	1
Changing clothes	7.500	2
Giving medicines	7.630	3
Dressing the wounds	6.556	7
Changing the “condom catheter”	6.688	5
Training the family members in simple nursing tasks	5.429	8
Others	6.667	6

Source: Primary data

**Table 10:** Type of psychological care provided by the volunteers

Type of psychological care	Mean	Rank
Chatting with the patients	4.786	1
Listening the sorrows and fears of patients	3.913	2
Listening to the concerns of the family members	3.400	3
Sharing of problems with patients and the family counselling	3.263	4

Source: Primary data

**Table 11:** Type of financial care provided by the volunteers

Type of financial care	Mean	Rank
Supply medicines at free of cost	4.966	1
Supply rice and provisions for the family	4.250	2
Provide wheel chairs/water beds, commodes, etc	4.143	3
Books, clothes, and school fees for the kids	3.000	4

Source: Primary data

**Table 12:** Type of spiritual care provided by the volunteers

Type of spiritual care	Mean	Rank
Psychological boost	9.759	1
Helped to establish/re-establish a sense of meaning	8.818	3
Encourage to reminisce with family and friends	7.0000	5
Prepare advance directives	8.778	4
Love and affection	8.917	2

Source: Primary data

All the patients in the age group of 20 to 40 years are highly satisfied with the services of the volunteers. Patients in the age group of above 40 years are either highly satisfied or satisfied with the services of the volunteers [Table 13]. There is no significant relationship between the age of the patients and their level of satisfaction in the services of the volunteers (Chi-square = 0.199 with 1 d.f. at 5% level [Table 13]). 56% of the patients are highly satisfied with the present medicines. Of which, 50% of them are in the age group of above 60 years and 32% of them are in the age group of 50 to 60 years [Table 14]. The majority of the patients in the age group of 40 to 50 years are satisfied with the medicines. Chi-square test result [Table 14] revealed that there is no significant relationship between the age of the patients and their level of satisfaction in the present medicines (Chi-square = 1.469 with 2 d.f. at 5% level).

**Table 13:** Age of the respondents and their level of satisfaction in the present services of the volunteers

Age	Highly satisfied	Satisfied	No opinion	Total
20-30	1			1
30-40	2			2
40-50	2	4		6
50-60	7	1	1	9
Above 60 years	22	3	7	32
Total	34	8	8	50

Source: Primary data; χ^2^ = 0.199 with 1 degree of freedom. Not significant at 5% level

**Table 14:** Age of the respondents and their level of satisfaction in the present medicines

Age	Highly satisfied	Satisfied	No opinion	Total
20-30	1			1
30-40	2	1		3
40-50	2	4	1	7
50-60	9	4		13
Above 60 years	14	11	1	26
Total	28	20	2	50

Source: Primary data; χ^2^ = 1.469 with 2 degrees of freedom; Not significant at 5% level

All the patients in the age group of 20 to 40 years are highly satisfied with the present medical treatment [Table 15]. Chi-square test result [Table 15] revealed that there is no significant relationship between the age of the patients and their level of satisfaction in the present medical treatment (Chi-square = 0.142 with 1 d.f. at 5% level). All the patients in the age group of 20 to 40 years are highly satisfied and patients in the age group of above 40 years are either highly satisfied or satisfied with the overall services of the units [Table 16]. Chi-square test result [Table 16] revealed that there is significant relationship between the age of the patients and their level of satisfaction in the overall services of the units (Chi-square = 3.907 with 1 d.f. at 5% level).

60% of the patients in the age group of above 60 years and 23% of the patients in the age group of 50 to 60 years have the opinion that the present services are qualitative and do not require any improvement [Table 17]. 31% of the patients in the age group of below 50 years and 69% of the patients in the age group of above 50 years demand for improvement in the quality of present palliative care services. Chi-square test result [Table 17] revealed that there is no significant difference in the opinion about the improvement in the present care among patients irrespective of their age (Chi-square = 2.973 with 1 d.f. at 5% level).

**Table 15:** Age of the respondents and their level of satisfaction in the present medical treatment

Age	Highly satisfied	Satisfied	Total
20-30	1		1
30-40	3		3
40-50	2	5	7
50-60	10	3	13
Above 60 years	16	10	26
Total	32	18	50

Source: Primary data; χ^2^ = 0.142 with 1 degree of freedom; Not significant at 5% level

**Table 16:** Age of the respondents and their level of satisfaction in the overall services

Age	Highly satisfied	Satisfied	Total
20-30	1		1
30-40	3		3
40-50	5	2	7
50-60	11	2	13
Above 60 years	15	11	26
Total	35	15	50

Source: Primary data; χ^2^ = 3.907 with1 degree of freedom. Significant at 5% level

**Table 17:** Age of the respondents and their opinion about improvement in the present care

Age	Yes	Percentage	No	Percentage	Total	Percentage
20-30	1	5			1	2
30-40	1	5	2	7	3	6
40-50	4	21	3	10	7	14
50-60	5	27	7	23	12	24
Above 60 years	8	42	19	60	27	54
Total	19	100	31	100	50	100

Source: Primary data; χ^2^ = 2.973 with1 degree of freedom. Not significant at 5% level

83% of the patients in the age group of above 50 years and 17% of the patients in the age group of 30 to 50 years do not require any services other than those offered by the units [Table 18]. However, 55% of the patients in the age group of above 50 years and 38% of them in the age group of 20 to 50 years need other services. Chi-square test result [Table 18] revealed that there is no significant relationship between the age of the patients and their need for other services (Chi-square = 1.941 with 1 d.f. at 5% level). 28% of the patients in the age group of above 50 years and 7% of the patients in the age group of 20 to 50 years feel that palliative care is very essential for the society. However, 74% of the patients in the age group of above 50 years and 26% of the patients in the age group of 40 to 50 years feel that these services as essential to the society [Table 19]. Chi-square test result [Table 19] revealed that there is no significant difference in the opinion about the need for PPC services in the society among patients of different age group (Chi-square = 0.549 with 1 d.f. at 5% level).

**Table 18:** Age of the respondents and their need for other care not provided by the unit

Age	Yes	Percentage	No	Percentage	Total	Percentage
20-30	1	11			1	2
30-40			3	7	3	6
40-50	3	27	4	10	7	14
50-60	4	44	9	22	13	26
Above 60 years	1	11	25	61	26	52
Total	9	100	41	100	50	100

Source: Primary data; χ^2^ = 1.941 with1 degree of freedom. Not significant at 5% level

**Table 19:** Age of the respondents and their opinion about the need for PPC services in the society

Age	Absolutely essential	Percentage	Essential	Percentage	Total	Percentage
20-30	1	3			1	2
30-40	3	9			3	6
40-50	3	9	4	26	7	14
50-60	11	30	2	13	13	26
Above 60 years	17	49	9	61	26	52
Total	35	100	15	100	50	100

Source: Primary data; χ^2^ = 0.549 with1 degree of freedom. Not significant at 5% level

57% of the female patients and 43% of male patients opined that palliative care service is absolutely essential for the society, whereas, 73% of male patients and 27% of female patients feel that these services are essential [Table 20]. Chi-square test result [Table 20] revealed that there is significant difference in the opinion about the necessity of PPC services in the society among the male and female patients (Chi-square = 3.907 with 1 d.f. at 5% level). After undergoing palliative care treatment, the pain suffered by the patients earlier could be reduced to a considerable extent, which they ranked as first. The hope of patients could be maintained or strengthened through palliative care treatment, which was ranked as second by the patients [Table 21]. It is understood that the services of the doctors are to be improved further by making available their services to all the palliative care patients in a uniform manner as and when the patients need it [Table 22]. Similarly, services of the nurses should also be improved, which was ranked as second by the patients.

**Table 20:** Sex of the respondents and their opinion about the necessity of PPC services in the society

Sex	Absolutely essential	Percentage	Essential	Percentage	Total	Percentage
Male	15	43	11	73	26	52
Female	20	57	4	27	24	48
Total	35	100	15	100	50	100

Source: Primary data; χ^2^ = 3.907 with1 degree of freedom; Significant at 5% level

**Table 21:** Type of relief to the patients after undergoing treatment

Type of relief after undergoing treatment	Mean	Rank
My hope is maintained/strengthened	12.0067	2
Pain is reduced	12.429	1
I feel more comfort	11.800	3
I am relieved from physical/mental suffering	10.571	7
I feel more secure	11.000	6
I feel relaxed	11.758	4
Feeling of independence	11.600	5
A good mental support system to help my family	8.800	11
The quality of my life is improved	9.429	9
I could get tremendous psychological boost	9.867	8
A great financial help to me and my family	9.000	10

Source: Primary data

**Table 22:** Areas where palliative care services are to be improved

Areas where services are to be improved	Mean	Rank
Services of the doctors	8.0000	1
Services of the nurses	7.667	2
Services of the volunteers	7.0000	4
Medicines	7.167	3
Increase in the frequency nurse’s visit	5.0000	5
Increase in the frequency of volunteer’s visit	7.0000	4

Source: Primary data

## CONCLUSION

In Ernakulam district, the majority of the patients under palliative care treatment are cancer patients in the age group above 60 years. It is revealed that the male patients who need palliative care outnumber the females. Volunteers play a major role in increasing the awareness of palliative care services among the community. After undergoing palliative care treatment, the pain suffered by the patients earlier could be reduced to a considerable extent and the hope of patients could be maintained or strengthened. It is understood that the services of the doctors and the nurses are to be improved further by making available their services to all palliative care patients in a uniform manner as and when the patients need it.

## Appendix: Impact of pain and palliative care services on patients

**Table:** Questionnaire

1.	Name of the patient	:								
2.	Name of district	:								
3.	Address	:								
4.	Phone No ________________								
5.	Name of panchayat	:								
6.	Ward No	:								
7.	Age	:	□ Less than 10 years	□ 10-20	□ 20-30	□ 30-40	□ 40-50	□ 50-60	□ 60 years and Above
			□ 40-50	□ 50-60	□ 60 years and Above			
8.	Weight of patient	:									
9.	Sex	:	□ M	□ F					
10.	Community	:	□ GEN	□ SC	□ ST	□ OBC				
11.	Marital status	:	□ Married	□ Single	□ Divorced/separated	□ Widow/Widower				
12.	Type of disease	:	□ Cancer	□ Burns	□ Spinal injuries	□ Osteoporosis	□ Arthritis	□ AIDS	□ Problems of old age and debility
			□ Psychiatric illness	□ Chronic Respiratory diseases	□ Chronic kidney disease	□ Chronic heart diseases	□ HIV	□ Accidents	
			□ Paraplegia or motor neuron diseases	□ Chronic Liver disease	□ Stroke	□ Others				
13.	How long have you been suffering from this disease?								
	□ Less than 6 months	□ 6-12 months	□ 12-18 months	□ 18-24 months	□ 24 months and More				
14.	Which system of medicine is predominantly followed?								
	□ Allopathy	□ Ayurveda	□ Homeopathy	□ Unnani	□ Siddha	□ Others (Specify)			
15.	Do you get pain relief from treatment	□ Yes	□ No								
16.	How long have you been undergoing palliative treatment?								
	□ Less than 6 months	□ 6-12 months	□ 12-18 months	□ 18-24 months	□ 24 months and More				
17.	How did you come to know about palliative care?								
	□ Volunteers	□ Hospital	□ Clinic	□ Doctor				
	□ Nurse	□ Media	□ Friends	□ Relatives	□ Others (Specify)				
18.	Please specify the type of care you get:								
	□ Home based care	□ Institution based Care	□ Both						
19.	If it is home-based care, please specify the form of care you get:								
	□ Community based care	□ Hospital Pain Clinic							
20.	Please specify the type of physical problems faced by you (Rank in the order of importance)								
	□ Pain	□ Breathlessness	□ Fatigue	□ Drowsiness					
	□ Insomnia	□ Dehydration	□ Constipation	□ Anorexia	□ Nausea					
	□ Physical losses	□ Edema	□ Incontinence	□ Loss of Function	□ Vomiting	□ Bed sores	□ Loss of Mobility/ Dependency		
	□ Fungating wounds	□ Disfigurement	□ Difficult to Swallow	□ Itching					
	□ Others (Specify)									
21.	Please specify the type of psychological problems faced by you (Mark with Distress Thermometer)								
	□ 0	□ 1	□ 2	□ 3	□ 4	□ 5	□ 6	□ 7	□ 8	□ 9	□ 10
22.	Please specify the type of social problem faced by you (Rank in the order of importance)									
	□ Not able to stay in my job /go to school	□ Social Isolation								
	□ Not able to fulfil my prior role in the family/society	□ Sadness								
	□ Not able to be active in the society/community	□ Depression								
	□ Lack of social safety	□ Fear	□ No relatives available for help							
	□ Neglect	□ Change in Faith/ Beliefs	□ Denial	□ Loss of Social Roles	□ Others (Specify)					
23.	Please specify the type of financial problems faced by you (Rank in the order of importance):									
	□ Poverty due to absence of income earning member in the family									
	□ Huge medical expenditure	□ Children dropped out of school								
	□ Intractable debt	□ Family member gave up work due to illness								
	□ Others (Specify)									
24.	Does any doctor visit you?	□ Yes	□ No.									
25.	If yes, please specify the periodicity of visit:									
	□ Regularly	□ Frequently	□ Occasionally							
	□ Only once	□ Not at all								
26.	Type of physical care provided by the doctor (Rank in the order of importance):									
	□ Medicines	□ Exercises and aids								
	□ Touch and closeness	□ Discussion between me and family members								
	□ Others (Specify)									
27.	Does any nurse visit you?	□ Yes	□ No.								
28.	If yes, please specify the periodicity of visit:									
	□ Regularly	□ Frequently	□ Occasionally							
	□ Only once	□ Not at all								
29.	Type of physical care provided by the nurses (Rank in the order of importance):									
	□ Bathing	□ Attending to the Bed Sore	□ Changing clothes							
	□ Giving medicines	□ Dressing the wounds	□ Changing the “Condom Catheter”							
	□ Training the family members in simple nursing tasks	□ Others (Specify)								
30.	Please specify the periodicity of visit of the volunteers:									
	□ Every day	□ Once in a week	□ Twice in a week							
	□ Thrice in a week	□ Once in a month	□ No periodicity							
31.	Type of physical care provided by the volunteers (Rank in the order of importance):									
	□ Bathing	□ Attending to the Bed Sore	□ Changing clothes							
	□ Giving medicines	□ Dressing the wounds	□ Changing the “Condom Catheter”							
	□ Training the family members in simple nursing tasks	□ Others (Specify)								
32.	Type of social care provided by the volunteers:									
	□ Supportive Counselling to the patient to face friends, neighbours and colleagues									
	□ Companionship	□ Others (Specify)								
33.	Type of psychological care provided by the volunteers (Rank on the basis of importance)									
	□ Chatting with the Patients	□ Listening the sorrows and fears of patients								
	□ Listening to the concerns of the family members	□ Sharing of problems with patients and the family counselling Others (Specify)								
34	Type of financial care provided by the volunteers (Rank in the order of importance):									
	□ Supply medicines at free of Cost	□ Supply rice and provisions for the family								
	□ Provide wheel chairs / Water beds, Commodes etc.									
	□ Books, Clothes, and school fees for the kids	□ Others (Specify)								
35.	Type of financial care provided by the volunteers (Rank in the order of importance):									
	□ Psychological boost	□ Love and Affection								
	□ Helped to establish/re-establish a sense of meaning and purpose to life									
	□ Encourage to reminisce with family and friends	□ Gift giving								
	□ Prepare advance directives	□ Assisting with life closure								
	□ Creation of legacies	□ Fulfilling the wishes	□ Others (Specify)							
36.	Type of relief you got after undergoing treatment (Rank in the order of importance):									
	□ My hope is maintained/strengthened	□ Pain is reduced	□ I feel more comfort							
	□ I am relieved from physical/mental suffering	□ I feel more secure								
	□ I feel relaxed	□ Feeling of Independence								
	□ I feel relaxed	□ Feeling of Independence								
	□ A good mental support system to help my family									
	□ The quality of my life is improved	□ I could get tremendous psychological boost	□ A great financial help to me and my family	□ Able to take food along with family members	□ Others (Specify)					
37.	Are you satisfied with the present services of the doctor of pain and palliative care unit?									
	□ Highly satisfied	□ Satisfied	□ Not satisfied	□ Dissatisfied	□ No opinion					
38.	Are you satisfied with the present services of the nurse of pain and palliative care unit?									
	□ Highly satisfied	□ Satisfied	□ Not satisfied	□ Dissatisfied	□ No opinion					
39.	Are you satisfied with the present services of the volunteers of pain and palliative care unit?									
	□ Highly satisfied	□ Satisfied	□ Not satisfied	□ Dissatisfied	□ No opinion					
40.	Are you satisfied with the present medical treatment provided by pain and palliative care unit?									
	□ Highly satisfied	□ Satisfied	□ Not satisfied	□ Dissatisfied	□ No opinion					
41.	Are you satisfied with the present medicines of pain and palliative care unit?									
	□ Highly satisfied	□ Satisfied	□ Not satisfied	□ Dissatisfied	□ No opinion					
42.	Are you satisfied with the overall services of the Unit?									
	□ Highly satisfied	□ Satisfied	□ Not satisfied	□ Dissatisfied	□ No opinion					
43.	Do you think that the?	□ Yes	□ No
44.	If yes, please specify the area where the services are to be improved (Rank in the order of importance):									
	□ Services of the doctors	□ Services of the nurses	□ Services of the volunteers							
	□ Medicines	□ Increase in the frequency of doctor’s visit	□ Increase in the frequency nurse’s visit	□ Increase in the frequency of volunteer’s visit						
	□ Others (specify)									
45.	Do you need any care other than the care provided by the unit?	□ Yes	□ No
46.	If yes, please specify the care needed by you:									
47.	Do you think that pain and palliative care is essential for patients suffering from terminal illness or old age problems?									
	□ Absolutely Essential	□ Essential	□ Somewhat essential	□ Not essential	□ No opinion					
48.	Please specify your suggestions if any:									
